# Health Literacy and Its Associations with Understanding and Perception of Front-of-Package Nutrition Labels among Higher Education Students

**DOI:** 10.3390/ijerph19148751

**Published:** 2022-07-19

**Authors:** Axelle Hoge, Mathilde Labeye, Anne-Françoise Donneau, Halehsadat Zahraei Nekoee, Eddy Husson, Michèle Guillaume

**Affiliations:** 1 Department of Public Health, University of Liège, 4000 Liege, Belgium; mathildelabeye@live.fr (M.L.); afdonneau@uliege.be (A.-F.D.); h.nekoee@uliege.be (H.Z.N.); eddy.husson@uliege.be (E.H.); mguillaume@uliege.be (M.G.); 2 Biostatistics and Research Method Center, University of Liège, University Hospital of Liège, 4000 Liege, Belgium

**Keywords:** front-of-package labels, nutrition labels, health literacy, students

## Abstract

*(1) Background*: Nutrition labels on the front of food packages have increasingly become the focus of research. However, too few studies have placed special emphasis on nutritionally at-risk subpopulations, such as young adults or those with low literacy/numeracy skills. The present study aimed to assess both the perception and objective understanding of three front-of-package labeling (FOPL) formats currently in use on the Belgian market, i.e., the Nutri-Score, Reference Intakes, and Multiple Traffic Lights, among students of varying health literacy (HL) levels. *(2) Methods*: A web-based survey was carried out among 2295 students of tertiary education in the province of Liège, Belgium. The questionnaire included questions related to general characteristics, objective understanding, and perception in response to the assigned FOPL format and level of HL. *(3) Results*: With respect to objective understanding, the Nutri-Score outperformed all other labels across all HL levels, and it was similarly understood in students of varying HL levels. Several students’ characteristics appeared to be associated with each cluster of perception, with the Nutri-Score cluster having the highest percentages of disadvantaged students, i.e., those with inadequate HL, from non-university institutions, with low self-estimated nutrition knowledge, and with low self-estimated diet quality. *(4) Conclusion*: Overall, the findings supported the Nutri-Score as particularly effective in guiding students in their food choices. Of particular importance is the fact that the summarized and graded color-coded nutritional label would be a useful strategy for those disadvantaged by limited HL.

## 1. Introduction

The European region is facing a rising threat imposed by obesity and non-communicable diseases (NCD), such as cardiovascular disease, diabetes, and some types of cancer. All of these diseases are closely related to unhealthy lifestyle practices, including a suboptimal diet [1].

The challenge today is to transform food systems such that they provide everyone with the healthy diets needed for optimal health and wellbeing [2,3]. High priority should be given to efforts that help citizens take responsibility for managing their own health and wellbeing, by making informed and healthier choices with regard to diet [4,5].

In this way, mandatory EU nutrition labeling is a key source of nutrition information to make informed choices (Regulation (EU) No 1169/2011). However, the back-of-pack nutritional information has been shown to be difficult to access, understand, and evaluate by consumers, particularly in populations at risk of limited health literacy (HL) [6,7].

Consequently, a number of countries are now introducing front-of-pack nutrition labeling (FOPL), the use of which has been recommended by the World Health Organization [8]. These FOP labels offer consumers at-a-glance information about the nutritional value of prepackaged products and promote healthier food choices at the point of purchase. A secondary objective of FOPL is to encourage the food industry to improve the nutritional composition of their products.

There is currently a wide variety of FOPL systems that have been implemented or are in the process of being created by public or private entities around the world. They can be classified into two main types: those that display numeric data on specific nutrient content (i.e., nutrient-specific schemes) and those that synthesize information on ingredient and/or nutrient content into a graphic and/or color-coded logos (i.e., summary schemes). While the implementation of FOP labels is currently voluntary, the European Commission recently underscored the need to implement a uniform and mandatory FOPL at the EU level [9]. In Belgium, the voluntary Nutri-Score FOPL system was officially adopted since 1 April 2019.

Evidence suggests that color-coded FOP labels (such as the Multiple Traffic Lights in England or the Nutri-Score in France) are more efficient at helping vulnerable populations better understand the nutritional quality of foods and make healthier food choices [10,11]. Beyond the common considerations of low education and low income, HL skills still need to be specifically addressed when the main performance features of the FOPL system are assessed [12]. The objective is to ascertain that the final graphical design is the best choice for the consumers of varying HL levels; otherwise, it may lead to adverse effects, with a potential increase in nutrition and health inequalities.

In that context, the aim of the present study was to assess both perception and objective understanding of three FOPL formats currently in use on the Belgian market (Nutri-Score, Reference Intakes, and Multiple Traffic Lights) in a population of particular interest. Adolescents and young adults experience a substantial share of the global NCD burden due to nutrition-related risk factors [13]. Tertiary education is a unique transition period, during which students have increasing independence and are developing food selection and preparation skills that can shape behaviors throughout life. Students are also more likely to increase snacking, decrease family meals, and consume more meals prepared outside the home [14,15,16]. Population-based nutrition interventions are needed to support healthy eating patterns during this relevant period of life [17,18].

This research provides insights into the potential effects of low literacy levels on the effectiveness of FOP labels in a population that needs immediate help to improve their diet quality in order to reduce health risks in the future.

## 2. Materials and Methods

### 2.1. Study Design and Population

A web-based survey, which took place between September 2019 and June 2020, was carried out among students of tertiary institutions, namely, universities, colleges, arts colleges, and social advancement education institutions, in the province of Liège, Belgium. The inclusion criteria were (1) being a tertiary-education student in the province of Liège, Belgium, (2) being at least 18 years old, and (3) being a French speaker.

Several methods of recruitment were applied. Out of the 27 identified institutions, 17 agreed to distribute the survey among their students through internal communication channels (emails, intranet, and/or website). Two reminders were mailed to the 17 institutions asking for the reactivation of their channels. Paper flyers were also posted in buildings across campuses. In addition, the social media pages of student organizations and groups were used to promote the survey. A total of 2781 students started the questionnaire, and 2295 (82.5%) completed it.

Participants gave their electronic consent before starting the survey. The study protocol was approved by the Ethics Committee of the Faculty of Medicine of the University of Liège, Belgium (approval reference: 2018/343).

### 2.2. Study Parameters and Measurement Instruments

The ad hoc online questionnaire was divided into four sections: (1) general characteristics, (2) objective understanding and (3) perception in response to the assigned FOPL format, and (4) HL.

#### 2.2.1. Characteristics of the Students

During the first part of the survey, participants provided sociodemographic, socioeconomic, and lifestyle information, including gender, age, nationality, type of higher education institution, field of education, smoking status, and household composition. Perceived financial resources were assessed via the following question: “In your opinion, do the financial resources you have allow you to meet your needs?” For analysis, the responses were dichotomized, whereby “with high difficulty” and “with fair difficulty” became “difficult”, while “very easily” and “fairly easily” became “easy”.

The students were invited to self-estimate and report their physical activity level by choosing one of the following options: “competitive sport” (intensive training and competitive sport more than once a week), “leisure-time activity” (such as jogging, cycling, walking, or gardening) at least 4 h per week, “leisure-time activity” less than 4 h per week, and “sedentary behaviors” (such as reading or watching TV).

Height and body weight were self-reported. Body mass index (BMI) was calculated using the standard formula of body weight in kilograms divided by the square of height in meters (kg/m^2^). Participants were classified into four categories according to definitions of the World Health Organization: underweight (BMI < 18.5 kg/m^2^), normal (BMI ≥ 18.5 to < 25 kg/m^2^), overweight (BMI ≥ 25 to < 30 kg/m^2^), and obese (BMI ≥ 30 kg/m^2^) [19].

With regard to nutritional knowledge, the participants were asked to self-estimate and report their level on a 10-point Likert scale (ranging from 0 “no knowledge” to 10 “excellent knowledge”). Other nutrition-related data comprised questions about grocery shopping frequency (always, sometimes, and never), the use of nutritional information during shopping (nutrition facts and ingredients), and self-estimated diet quality.

#### 2.2.2. FOPL Formats Tested

As noted before, three FOPL formats providing varying levels of information about the products’ nutritional quality were tested in the present study. In the introduction to the questionnaire, the different label formats were briefly introduced to the students.

Nutrient-specific schemes were as follows:

1. Reference Intakes (RI), formerly called Guideline Daily Amounts (GDA). This label displays energy, sugar, (saturated) fat, and salt content per 100 g/mL and per portion of a certain product, as well as contributions to recommended daily amounts (in percentages). This label can be found on most food packaging on the Belgian market, based on a voluntary initiative from manufacturers. The RI was generated using the Food and Drink Industry’s guidance [20].
2. Multiple Traffic Lights (MTL). This label displays energy, sugar, (saturated) fat, and the salt content of food in red, amber, or green according to a set threshold. The criteria of the FSA were applied to assign the color codes [21].

The summary scheme was as follows:

1. Nutri-Score. This label rates the overall nutritional quality of a given food item with five colors/letters from red/E (least healthy) to green/A (most healthy). The Nutri-Score is calculated per 100 g/mL, taking into account energy, saturated fat, sugar, sodium, fiber, protein, and proportion of fruit, vegetables, and nuts. The Nutri-Score was generated on the basis of Santé Publique France guidance [22].

FOPL formats were not associated with any other visible nutritional information or claims. A situation with no FOPL on the food package was used as a reference.

#### 2.2.3. Objective Understanding

The procedure was developed on the basis of previous publications [23,24]. Objective understanding of the three different FOP labels was assessed under four different conditions: three alternatives corresponding to the three different FOP labels and one alternative with no label. Subjects were asked to rank three products belonging to the same food category according to their nutritional quality. Specifically, participants were shown pictures of the three products, each featuring the respective FOP label, and they were asked to complete the following task: “From your point of view, please rank these products according to their nutritional quality.” For the ranking, participants could choose among the following options: “lowest nutritional quality”, “intermediate nutritional quality”, or “highest nutritional quality”. An option of “I do not know” was also proposed for each food category.

Four different food categories were selected for testing, namely, pizzas, regular dairy products, breakfast cereals, and appetizers (crisps and peanuts), because they are frequently consumed in Belgium [25,26,27] at various occasions (breakfast meals, ready-to-eat lunch/dinner meals, and desserts), and they exhibit sufficient nutritional variability within a category. To achieve a realistic setting, we identified real-world products of relatively low, intermediate, and high nutritional quality in each food category following an examination of food composition databases. A nutritional quality rating was assigned on the FOP afterward, and any other visible nutritional information or claims were removed.

To avoid the potential effects of the product category upon understanding of the FOP label (i.e., due to knowledge of specific products), each label was associated with all product categories. Each participant was shown four label/product combinations where all four FOP label conditions and product categories were represented. A total of 16 different versions of the questionnaire were then used. For example, one participant was shown the Nutri-Score on appetizers and the RI on breakfast cereals, while another participant was shown the RI on appetizers and the MTL on breakfast cereals.

The choice of combination was made at random. However, meters were applied to ensure that an equal number of men and women were shown each label/product category combination while controlling for the potential order effects of the labels.

Ranking was considered correct if the three products were ranked in the expected order (i.e., according to information on nutritional quality provided by the labels). Ranking was considered incorrect if at least one mistake was made or if the answer “I do not know” was given.

#### 2.2.4. Perception

Label acceptability was evaluated using French survey items which have already been applied to FOP labels by Ducrot et al. [23,28]. Overall, 13 questions were asked on various aspects of liking (e.g., “This is my preferred FOP label”), attractiveness (e.g., “This FOP label provides reliable information”), and perceived cognitive workload (e.g., “This label is too complex for understanding”) (see Supplementary Table S1). For each question, subjects were asked to select, among the three formats, the label that best corresponded to the proposed statements (one possible answer). The participants could also select that “none” of the proposed labels corresponded to their perception.

#### 2.2.5. Health Literacy

In the final part of the survey, participants were asked to self-report HL using the European Health Literacy Survey Questionnaire, short French-speaking version with 16 items (HLS-EU-Q16), developed by the HLS-EU Consortium [29,30,31]. The tool measures overall HL considering its four dimensions (the way people access, understand, appraise, and apply health information) in three broad domains of health (healthcare, disease prevention, and health promotion).

Each of the 16 items was scaled on a four-point Likert ranging from “very difficult” to “very easy”. According to the guidelines, the responses were dichotomized into “difficult” (scored with 0) and “easy” (scored with 1). Summing these responses gave an HLS-EU-Q16 final score ranging from 0 (low/no HL) to 16 (high HL). Missing responses were not allowed. Three levels were defined as recommended: inadequate HL (0–8), problematic HL (9–12), and sufficient HL (13–16).

### 2.3. Statistical Analysis

Analyses were performed on participants who had completed the four parts of the survey. The students who responded “I do not know” to three or more food categories in the “objective understanding” questionnaire were excluded from statistical analysis. Self-estimated nutrition knowledge and diet quality were split at the median to form high and low groups before analysis.

Medians and interquartile ranges (IQRs) were presented for quantitative variables, while qualitative variables were summarized with frequencies and percentages. Chi-square tests were performed to compare the distribution of subjects with inadequate, problematic, and sufficient HL with respect to sociodemographic, socioeconomic, and lifestyle variables.

Univariate and multivariate mixed logistic regression models with random intercepts were implemented to explore how FOP labels, HL, and other students’ characteristics were associated with objective understanding. Variables displaying a significance level of *p* < 0.1 in univariate models were considered in the multivariate model. Results were expressed in terms of an odds ratio (OR) with a 95% confidence interval (95% CI). The associations were considered statistically significant if *p*-values were <0.05.

In addition, in order to explore label performances across subgroups at risk, univariate adjusted mixed logistic regression models with random intercepts were fitted to evaluate how the four FOPL situations (Nutri-Score, Reference Intakes, Multiple Traffic Lights, and none) and students’ characteristics affected the probability of a correct answer.

In the cluster analysis process, the number of clusters was determined by hierarchical clustering and the “NbClust” package of 30 indices for determining the relevant number of clusters. According to this approach, the majority of the indices were emphasized to consider four clusters. Then, K-means was applied for assigning clusters to subjects. The derived cluster results were evaluated using original FOPL format performance, and then four clusters were labeled as one of the no-label reference situations, Nutri-Score, Guideline Daily Amounts, and Multiple Traffic Lights according to the highest level of consistency. Lastly, univariate multinomial logistic regression models were applied to explore the association between HL, students’ characteristics, and the cluster results.

Statistical analyses were conducted using R studio.

## 3. Results

### 3.1. Characteristics of the Sample

A total of 2295 students completed the entire survey. Among them, 35 (1.5%) were excluded because they responded “I do not know” to three or more food categories in the “objective understanding” questionnaire. In total, 2260 students were available for the analysis, of which 75% were women, 65% pursued their tertiary-level education in a university, and 57% studied in non-health-related fields such as art, human and social sciences, and science and technology.

Almost half of the participants lived with their family, and 19% reported they always used nutrition information when shopping (Table 1).

With regard to HL, the median (IQR) score was 12 (9–13). Approximately 63% of students had limited HL, including 16% with inadequate HL and 47% with problematic HL. It appears from Table 1 that HL was significantly related to sex, age, higher education institution, field of education, physical activity, perceived financial resources, self-estimated nutrition knowledge, grocery shopping frequency, use of nutritional information during shopping, and self-estimated diet quality. Indeed, the proportions of men, older and university students, students in health-related fields, and individuals with higher physical activity were higher within the sufficient HL group compared to the insufficient and limited HL groups.

### 3.2. Objective Understanding

#### 3.2.1. Influence of FOPL Formats, HL, and Students’ Characteristics on the Ability to Rank Products According to Nutritional Quality

Results showing the association between objective understanding and FOPL formats, HL, and students’ characteristics are presented in Table 2. In both univariate and multivariate models, compared to the no-label reference situation, the odds of ranking products correctly according to the nutritional quality was increased for all FOP labels tested. Among the three FOPL formats, Nutri-Score achieved the highest performance, followed by MTL and RI. More specifically, students were 15 times more likely to rank products correctly when Nutri-Score was provided on the fronts of packages (OR 15.34 (95% CI 13.09–17.96)).

Data are presented as the OR (95% CI). Boldface indicates statistical significance (*p* < 0.05).

The ability to rank products according to nutritional quality did not significantly differ in any HL level when compared to inadequate HL. Regarding general characteristics, from a multivariate standpoint, students from university institutions (OR 1.16 (95% CI 1.05–1.29)) and those with higher perceived nutrition knowledge (OR 1.16 (95% CI 1.04–1.29)) provided more correct responses on the product-ranking task than did those in the reference group.

#### 3.2.2. Comparison of FOPL Format Performance across Subgroups

When studying FOPL format performance across subgroups of students (Table 3), we observed the same trend whatever the subgroup. Compared to the no-label reference situation, Nutri-Score conferred the greatest odds for ranking products correctly according to the nutritional quality in all subgroups (lowest OR 11.47 (95% CI 8.10–16.23) among students reported always using nutritional information when shopping; greatest OR 39.83 (95% CI 17.29–91.73) among obese subjects). It was always followed by MTL (lowest OR 2.49 (95% CI 1.47–4.24) among former smokers; greatest OR 5.08 (95% CI 2.66–9.71) among obese subjects) and then by RI (lowest OR 0.93 (95% CI 0.61–1.42) among thin subjects; greatest OR 2.59 (95% CI 1.36–4.92) among obese subjects).

The presence of Nutri-Score was characterized by the highest increase in correct ranking compared to the “no-label” situation in appetizer, dairy product, and pizza categories, but not in breakfast cereals. When testing the latter food category, MTL achieved the highest performance (OR 16.51 (95% CI 12.31–22.13)).

### 3.3. Perception

The distribution of the responses to each question related to the perception of FOP labels is displayed in Supplementary Table S2. Overall, perceptions of students were in favor of all FOPL formats. The Nutri-Score specifically was the label receiving the highest number of responses on positive perception dimensions, followed by the MTL. Conversely, the RI was the most frequently selected with regard to negative perception dimensions (judgment, complexity, and time to process). Thirty-five percent of participants considered that none of the labels proposed were guilt laden.

Lastly, students were assigned into four clusters according to their perception of FOP labels. Clusters represented 40.0% (crude *n* = 904), 26.7% (crude *n* = 604), 19.7% (crude *n* = 446), and 13.5% (crude *n* = 306) of participants.

Nutri-Score and Multiple Traffic Lights won the most students’ favor in the clusters that bore their names, especially regarding the following aspects: the label allowing them to choose healthier products, label wanted on the FOP, preferred label, and trustworthiness. In its respective cluster, the preference for the Reference Intakes was prominent for certain aspects in particular. For example, 59.6% of students considered that the RI provided all the information needed, 54.7% considered it trustworthy, and 44.8% considered that the RI provided reliable information. Conversely, the grouping of the cluster labeled “none” and the distinction with the others were less clear. In the latter cluster, we observed the highest percentage of students who considered that none of the presented FOP labels was their favorite (20.3%), was trustworthy (18.6%), or provided reliable information (15.4%); however, responses were somewhat less marked. The mapping of perception responses across clusters is included in the Supplementary Materials (Supplementary Table S3).

The results of the multivariable-adjusted HL and students’ characteristics according to the clusters are shown in Table 4. From this table, it can be observed that participants who had inadequate HL were significantly more frequent in the Nutri-Score cluster, while participants who had sufficient HL were significantly more frequent in the “none” and RI clusters (*p* = 0.022). The proportion of older students was higher in the “none” cluster (36.9%), while that of younger students was higher in the RI (52.9%) and Nutri-Score (51.3%) clusters. Non-university students were more common in the Nutri-Score cluster, whereas university students were more common in the MTL cluster (*p* < 0.0001). Lastly, participants who reported always using nutrition information when shopping were more prevalent in the “none” cluster, while those who reported never using this kind of information when shopping were more prevalent in the Nutri-Score cluster.

## 4. Discussion

Nutrition labels on the front of food packaging have increasingly become the focus of research. There are existing data on the use and effectiveness of FOPL in Belgium [32], France [23], the Netherlands [33], and many other European countries [11]. However, too few studies have placed special emphasis on nutritionally at-risk subpopulations, such as young adults or those with low literacy/numeracy skills. This research aimed to shed light on this important component of the evaluation processes.

Poor HL among the population has emerged as an underestimated public health problem globally [34]. A growing number of studies have indicated that people with low HL are more likely to have poorer health statuses and engage in harmful health behaviors, which in turn puts them at a higher risk of a shorter life expectancy [35,36]. In particular, low literacy skills would interfere with the pathway of behaviors leading to a healthy diet, such as judging portion size, accessing nutritional information, or understanding and utilizing nutritional labels [37,38,39].

In Europe, the European Health Literacy Survey revealed that 12% of adults have inadequate HL, while 35% have limited HL [31]. The Belgian situation is quite similar, with around 40% of the adults having inadequate or problematic HL [40,41]. Our study showed a higher prevalence, with 16% and 47% of students having inadequate and problematic HL, respectively. These results are in line with previous studies when the young adult population was specifically addressed. According to a recent systematic review, university students tend to have lower HL scores compared to reference samples [42]. Respectively, 20.1% and 41.4% of European students were ranked as having inadequate and problematic HL using the HLS-EU-Q16 [43]. In Belgium, the 18–24 age group had the highest insufficient HL score (22.4%) compared to the other age categories [40].

Among the determinants presented, our results provided support for relationships among HL and sex, age, education degree, field of education, physical activity, and socioeconomic status, as previously reported [42]. Young adults, who have been highlighted as a specific group regarding HL and its determinants throughout the literature [42,44], could benefit from FOP labels for their own health. Recently, the “High in” FOPL was shown to be significantly more effective than current regulated labeling in helping Canadian consumers of varying HL levels to identify foods high in nutrients related to public health concerns [45].

Our study showed that all FOP labels seemed to improve students’ capacities to understand the nutritional quality of products; however, among the available options, the Nutri-Score was the highest-performing label in this task. We also observed that some students’ subgroups were more likely to accurately interpret FOP labels. Educational level and nutrition knowledge were general characteristics affecting the likelihood of understanding nutrition labels, which is consistent with previous studies in the area [6,24,46].

Students’ general characteristics were found not to affect the performance of the summarized and graded color-coded label. Across student subgroups, the Nutri-Score had the strongest positive association with participants’ ability to rank products, followed by MTL and RI. This trend was not applicable to all product categories, since, in the breakfast cereal category, the MTL was the best performer, followed by the Nutri-Score and RI.

In industrialized countries, diet disparities have been widely pointed out in the general population, as well as, to a lesser extent, in adolescents and young adults [47]. These latter groups represent a population at an elevated risk of low levels of physical activity, an increased risk of weight gain, and poor dietary patterns [48]. This pivotal stage of young adulthood, where lifelong health behaviors are heavily influenced or fostered, requires specific public health nutrition strategies taking into account social disparities behind health and nutrition inequalities [11,47]. Interestingly, the results of the present study highlighted the fact that the Nutri-Score outperformed all other labels across all HL levels, and the summarized and graded color-coded label was similarly understood in students of varying HL levels.

Overall, our findings are in line with previous work on the effectiveness of the Nutri-Score in the general population. An international comparative experimental study conducted across 12 countries concluded that, among the tested labels, the Nutri-Score was the most efficient in facilitating a consumer’s comprehension of foods’ nutritional quality [49]. Another study among a convenience sample of 1007 Belgian consumers was also favorable toward the Nutri-Score, compared to Health Star Ratings, MTL, GDA, and Warning symbols [32]. There is evidence supporting the positive impact of nutrition labels on students in their emerging adulthood years [50]. However, to our knowledge, very few studies have looked at the effects of different FOPL formats, currently implemented in different countries internationally [51,52,53]. An experimental study found that changes in purchase intentions on campus were not affected by the nature of FOP labels used (i.e., MTL and RI) [54]. More recently, the Nutri-Score was demonstrated to have benefits on the healthier purchasing intentions of students [55].

There are several factors that strongly affect the tendency by consumers to use FOPL. These include respondents’ trust in the label provided, appealing label design and comprehensible format, and the time needed to process the information. In terms of perception, our study showed that the Nutri-Score and MTL were well accepted by the participants; however, the RI was perceived as “being complex to understand” with a longer “time to process” to a greater extent than the two other labels. In addition, it was observed that students with inadequate HL gave preference to the Nutri-Score, those with problematic HL gave preference to the MTL, and those with sufficient HL gave preference to the RI or no-label situation. Each type of FOPL has strengths and weaknesses [56], some of which provide valuable insights into these results. Students with inadequate HL face the greatest challenges in accessing, understanding, and evaluating health/nutrition labelling information. Their preference for an overall summary system score (e.g., the Nutri-Score) can be explained by the fact that it provides them with a high level of guidance about the nutritional quality of a food product. The MTL is a hybrid scheme providing a mix of factual information and interpretative elements, which may have attracted the preference of students with problematic HL. Our results showed that the MTL was specifically the label receiving the highest number of responses on the following positive perception dimension: “This label provides me with the information I need” (Supplementary Table S2). This label may allow students to pay attention to particular nutrients of concern/interest. However, consumers may have difficulty identifying the healthiest options when there is a tradeoff between nutrients [56]. Lastly, it was observed that students with sufficient HL were more frequent in preference clusters “none” (41.2%) or RI (39.5%). This may reflect their greater abilities to process factual health/nutritional information provided by nutrition fact labels or RI. It is important to remember that, although interesting, findings on FOPL perception do not reflect its good understanding (objective understanding) or its real impact on food purchases or dietary quality.

Several students’ characteristics appeared to be associated with each preference cluster, with the Nutri-Score cluster concentrating the highest percentages of disadvantaged students, i.e., those with inadequate HL, from non-university institutions, with low self-estimated nutrition knowledge and low self-estimated diet quality.

Among the strengths of this study, we can highlight its large sample size, especially considering the challenges of research recruitment in a higher education setting [57]. In addition, we used different recruitment methods at different times in the academic year in order to mobilize different student profiles from different tertiary institutions. With regard to objective understanding, all combinations of food categories and FOP labels were tested to control for the potential bias of food products. Lastly, the use of the HLS-EU-Q16 reduced the risk of overlap when studying HL and FOPL understanding, which can exist when using the Newest Vital Sign [38].

Limitations should also be acknowledged. First, body weight and height were self-reported by students. However, we observed that our participants had anthropometric features similar to Belgian young adults aged between 18 and 24 years old [58]. The median (IQR) BMI was 22.0 (20.1–24.5) kg/m^2^ in our sample vs. 21.7 (20.0–24.3) kg/m^2^ in the reference population. Furthermore, 8.7% of our students were classified as underweight, 16.0% were classified as overweight, and 5.0% were classified as obese vs. 6.0%, 18.0%, and 6.4% of Belgian young adults, respectively. Secondly, students who participated in the survey may have had a greater interest in the subject matter and, therefore, may have been more health- and nutrition-conscious. On the one hand, we observed that there were more self-reported never smokers (77.8% vs. 58.6%) than in Belgian young adults [59]. On the other hand, the overrepresentation of females in our sample is consistent with what is observed in European higher education (where at least 60% of students are women) [60]. In addition, as reported above, we observed that our participants had anthropometric features similar to the reference population. The HL profile of students was also in line with previous findings. Additionally, our results did not show a statistically significant association between HL and BMI as a health-proxy variable. Thirdly, we used real-world food products, which may have led to brand-related effects on the results (e.g., brand loyalty, habit, and preference).

## 5. Conclusions

To conclude, this study is one of the first to provide insights into the relationship between varying HL levels with respect to the understanding and perception of FOPL formats in an under-investigated population. Overall, the findings supported the Nutri-Score as particularly effective in guiding students in their food choices. Of particular importance is the fact that its performance in ranking products according to nutritional quality and its perception were not affected by HL levels, which makes the Nutri-Score a useful strategy for those students disadvantaged by limited HL. Additional evidence is required from studies testing FOPL on food purchases in virtual or real food-shopping environments, including a higher education setting.

## Figures and Tables

**Table 1:** Descriptive statistics of students’ characteristics globally and according to HL levels (*n* = 2260).

Variable	All (*n* = 2260)	Inadequate HL (*n* = 358)	Problematic HL (*n* = 1075)	Sufficient HL (*n* = 827)	*p*-Value ^a^
Sex					**<0.0001**
Men	563 (24.9)	54 (15.1)	269 (25.0)	240 (29.0)	
Women	1697 (75.1)	304 (84.9)	806 (75.0)	587 (71.0)	
**Age (years)**					**<0.0001**
≤21	1075 (47.6)	190 (53.1)	543 (50.5)	342 (41.4)	
22–23	554 (24.5)	86 (24.0)	270 (25.1)	198 (23.9)	
≥24	631 (27.9)	82 (22.9)	262 (24.4)	287 (34.7)	
**Nationality**					0.085
Belgian	1901 (84.1)	313 (87.4)	907 (84.4)	681 (82.3)	
Others	359 (15.9)	45 (12.6)	168 (15.6)	146 (17.7)	
**Higher education institution**					**<0.0001**
Non-university	788 (34.9)	153 (42.7)	394 (36.7)	241 (29.1)	
University	1472 (65.1)	205 (57.3)	681 (63.3)	586 (70.9)	
**Education field**					**<0.0001**
Health	978 (43.3)	118 (33.0)	436 (40.6)	424 (51.3)	
Others	1282 (56.7)	240 (67.0)	639 (59.4)	403 (48.7)	
**Smoking Status**					0.070
Current smoker	382 (16.9)	74 (20.7)	186 (17.3)	122 (14.8)	
Former smoker	120 (5.3)	21 (5.9)	49 (4.6)	50 (6.0)	
Never smoker	1758 (77.8)	263 (73.5)	840 (78.1)	655 (79.2)	
**Physical activity**					**<0.001**
Competitive sport	270 (11.9)	29 (8.1)	144 (13.4)	97 (11.7)	
Leisure-time activity ≥4 h/week	619 (27.4)	84 (23.5)	296 (27.5)	239 (28.9)	
Leisure-time activity <4 h/week	951 (42.1)	155 (43.3)	432 (40.2)	364 (44.0)	
Sedentary behavior	377 (16.7)	85 (23.7)	181 (16.8)	111 (13.4)	
**BMI (kg/m^2^)**					0.275
≤18.5	196 (8.7)	23 (6.4)	103 (9.6)	70 (8.5)	
18.5–24.9	1583 (70.0)	257 (71.8)	745 (69.3)	581 (70.3)	
25.0–29.9	362 (16.0)	53 (14.8)	176 (16.4)	133 (16.1)	
≥30.0	112 (5.0)	25 (7.0)	47 (4.4)	40 (4.8)	
**Household composition**					**0.213**
Alone	320 (14.2)	50 (14.0)	154 (14.3)	116(14.0)	
With family	1124 (49.7)	190 (53.1)	546 (50.8)	388 (46.9)	
Shared house	816 (36.1)	118 (33.0)	375 (34.9)	323 (39.1)	
Perceived financial resources					<0.0001
Difficult	557 (24.6)	117 (32.7)	267 (24.8)	173 (20.9)	
Easy	1703 (75.4)	241 (67.3)	808 (75.2)	654 (79.1)	
**Self-estimated nutrition knowledge**					**<0.0001**
Low level	1132 (50.1)	221 (61.7)	586 (54.5)	325 (39.3)	
High level	1128 (49.9)	137 (38.3)	489 (45.5)	502 (60.7)	
**Grocery shopping frequency**					**0.002**
Always	845 (37.4)	109 (30.4)	397 (36.9)	339 (41)	
Sometimes	802 (35.5)	130 (36.3)	375 (34.9)	297 (35.9)	
Never	613 (27.1)	119 (33.2)	303 (28.2)	191 (23.1)	
**Use of nutritional information during shopping**					**<0.001**
Always	437 (19.3)	55 (15.4)	185 (17.2)	197 (23.8)	
Sometimes	1269 (56.2)	208 (58.1)	602 (56)	459 (55.5)	
Never	554 (24.5)	95 (26.5)	288 (26.8)	171 (20.7)	
**Self-estimated diet quality**					**<0.0001**
Low quality	1161(51.4)	221 (61.7)	577 (53.7)	363 (43.9)	
High quality	1099 (48.6)	137 (38.3)	498 (46.3)	464 (56.1)	

Data are presented as numbers (%). ^a^ *p*-Values based on chi-square test. Boldface indicates statistical significance (*p* < 0.05).

**Table 2:** Univariate and multivariate mixed logistic regression models showing the association between objective understanding and FOPL formats, HL, and other students’ characteristics.

	Univariate Model			Multivariate Model		
Variable	OR (95% CI)	*p*-Value	Global *p*-Value	OR (95% CI)	*p*-Value	Global *p*-Value
**FOPL formats**			**<0.0001**			**<0.0001**
None	1.00			1.00		
Nutri-Score	15.31 (13.07–17.93)	**<0.0001**		15.34 (13.09–17.96)	**<0.0001**	
RI	1.28 (1.13–1.46)	**0.0001**		1.28 (1.13–1.46)	**0.0001**	
MTL	3.35 (2.95–3.81)	**<0.0001**		3.35 (2.95–3.81)	**<0.0001**	
**Health literacy**			0.081			0.439
Inadequate HL	1.00			1.00		
Problematic HL	1.03 (0.91–1.16)	0.647		1.01 (0.87–1.15)	0.98	
Sufficient HL	1.12 (0.99–1.27)	0.064		1.07 (0.91–1.23)	0.43	
**Sex**			0.646			
Women	1.00					
Men	1.02 (0.93–1.12)	0.646				
**Age (years)**			0.154			
≤21	1.00					
22–23	0.98 (0.89–1.09)	0.754				
≥24	1.09 (0.98–1.20)	0.094				
**Nationality**			0.672			
Others	1.00					
Belgian	1.02 (0.91–1.15)	0.672				
**Higher education institution**			**0.004**			**0.007**
Non-university	1.00			1.00		
University	1.13 (1.04–1.24)	**0.004**		1.16 (1.05–1.29)	**0.007**	
**Education field**			0.065			0.413
Others	1.00			1.00		
Health	1.08 (0.99–1.17)	0.065		1.04 (0.94–1.15)	0.413	
**Smoking status**			0.903			
Current smoker	1.00					
Former smoker	0.97 (0.79–1.20)	0.804				
Never smoker	1.01 (0.91–1.13)	0.820				
**Physical activity**			0.584			
Sedentary behavior	1.00					
Competitive sport	1.08 (0.92–1.26)	0.336				
Leisure-time activity ≥ 4 h/week	1.09 (0.96–1.24)	0.176				
Leisure-time activity < 4 h/week	1.06 (0.94–1.19)	0.367				
**BMI (kg/m^2^)**			0.171			
≤18.5	1.29 (1.02–1.63)	0.030				
18.5–24.9	1.20 (0.99–1.45)	0.063				
25.0–29.9	1.23 (0.99–1.52)	0.057				
≥30.0	1.00					
**Household composition**			0.138			
Alone	1.00					
With family	0.97 (0.86–1.10)	0.625				
Shared house	1.06 (0.93–1.21)	0.359				
**Perceived financial resources**			0.142			
Difficult	1.00					
Easy	1.07 (0.98–1.18)	0.142				
**Self-estimated nutrition knowledge**			**<0.0001**			**0.007**
Low level	1.00			1.00		
High level	1.18 (1.08–1.28)	**<0.0001**		1.16 (1.04–1.29)	**0.007**	
**Grocery shopping frequency**			0.021			0.067
Always	1.14 (1.03–1.26)	**0.013**		1.13 (1.00–1.29)	0.056	
Sometimes	1.14 (1.03–1.27)	**0.014**		1.15 (1.01–1.30)	**0.032**	
Never	1.0			1.00		
**Use of nutritional information during shopping**			0.191			
Always	1.10 (0.97–1.25)	0.123				
Sometimes	1.09 (0.98–1.20)	0.097				
Never	1.0					
**Self-estimated diet quality**			**0.003**			0.175
Low quality	1.00			1.00		
High quality	1.13 (1.04–1.23)	**0.003**		1.07 (0.97–1.19)	0.175	

**Table 3:** Mixed logistic regression models showing the association between objective understanding and FOP labels, across subgroups at risk.

Variables	Nutri-Score (vs. No Label)	Reference Intakes (vs. No Label)	Multiple Traffic Lights (vs. No Label)	Global *p*-Value
	OR (95% CI)	OR (95% CI)	OR (95% CI)	
**Product category**				
Appetizers	42.94 (30.23–61.00)	1.29 (0.89–1.87)	1.12 (0.76–1.64)	**<0.0001**
Breakfast cereals	13.92 (10.44–18.56)	3.71 (2.86–4.83)	16.51 (12.31–22.13)	**<0.0001**
Dairy products	13.57 (9.78–18.82)	0.97 (0.77–1.23)	3.29 (2.58–4.20)	**<0.0001**
Pizzas	13.44 (9.48–19.04)	0.71 (0.56–0.90)	2.61 (2.05–3.34)	**<0.0001**
**Health literacy**				
Inadequate HL	14.70 (13.05–18.97)	1.17 (1.01–1.35)	3.18 (2.75–3.68)	**<0.0001**
Problematic HL	14.61 (11.67–18.29)	1.40 (1.17–1.69)	3.37 (2.81–4.06)	**<0.0001**
Sufficient HL	15.88 (12.17–20.73)	1.25 (1.01–1.54)	3.41 (2.77–4.21)	**<0.0001**
Sex				
Men	14.45 (10.60–19.71)	1.68 (1.30–2.17)	3.93 (3.03–4.10)	**<0.0001**
Women	15.70 (13.05–18.97)	1.17 (1.01–1.35)	3.18 (2.75–3.68)	**<0.0001**
**Age (years)**				
≤21	17.09 (13.54–21.57)	1.30 (1.08–1.57)	3.48 (2.89–4.18)	**<0.0001**
22–23	14.98 (10.94–20.52)	1.34 (1.04–1.73)	3.03 (2.35–3.90)	**<0.0001**
≥24	13.02 (9.68–17.51)	1.20 (0.95–1.53)	3.47 (2.72–4.44)	**<0.001**
**Nationality**				
Belgian	14.41 (12.14–17.09)	1.22 (1.07–1.41)	3.16 (2.75–3.63)	**<0.0001**
Others	21.58 (14.21–32.77)	1.66 (1.19–2.30)	4.66 (3.34–6.49)	**<0.0001**
**Higher education institution**				
University	14.17 (11.66–17.20)	1.24 (1.06–1.45)	3.19 (2.73–3.73)	**<0.0001**
Non-university	17.83 (13.57–23.44)	1.37 (1.10–1.71)	3.71 (2.97–4.62)	**<0.0001**
**Education field**				
Health	16.39 (12.80–20.99)	1.24 (1.03–1.50)	3.15 (2.60–3.82)	**<0.0001**
Others	14.64 (11.91–17.99)	1.31 (1.11–1.56)	3.51 (2.96–4.16)	**<0.0001**
**Smoking status**				
Current smoker	18.08 (12.18–26.83)	1.61 (1.18–2.20)	3.79 (2.76–5.22)	**<0.0001**
Former smoker	15.17 (7.87–29.21)	1.35 (0.79–2.31)	2.49 (1.47–4.24)	**<0.001**
Never smoker	14.92 (12.47–17.84)	1.22 (1.05–1.41)	3.35 (2.89–3.87)	**<0.0001**
**Physical activity**				
Competitive sport	12.70 (8.17–19.74)	1.20 (0.84–1.71)	2.93 (2.05–4.19)	**<0.001**
Leisure-time activity ≥4 h/week	16.42 (12.02–22.44)	1.25 (0.98–1.60)	3.69 (2.88–4.75)	**<0.001**
Leisure-time activity <4 h/week	15.34 (12.03–19.56)	1.28 (1.05–1.55)	3.11 (2.57–3.77)	**<0.0001**
Sedentary behavior	15.22 (10.38–22.31)	1.39 (1.02–1.91)	3.62 (2.64–4.96)	**<0.0001**
**BMI (kg/m^2^)**				
≤18.5	17.91 (10.10–31.76)	0.93 (0.61–1.42)	3.20 (2.10–4.88)	**<0.001**
18.5–24.9	13.63 (11.34–16.39)	1.34 (1.15–1.56)	3.23 (2.78–3.76)	**<0.0001**
25.0–29.9	20.25 (13.21–31.03)	1.05 (0.76–1.46)	3.69 (2.67–5.11)	**<0.001**
≥30.0	39.83 (17.29–91.73)	2.59 (1.36–4.92)	5.08 (2.66–9.71)	**<0.0001**
**Household composition**				
Alone	14.78 (9.78–22.33)	1.47 (1.05–2.07)	4.21 (2.98–5.94)	**<0.0001**
With family	14.76 (11.83–18.42)	1.24 (1.03–1.48)	3.37 (2.81–4.04)	**<0.0001**
Shared house	16.54 (12.61–21.70)	1.28 (1.04–1.58)	3.05 (2.47–3.76)	**<0.0001**
**Perceived financial resources**				
Difficult	14.87 (10.82–20.43)	1.16 (0.89–1.50)	3.05 (2.36–3.95)	**<0.001**
Easy	15.46 (12.88–18.55)	1.32 (1.14–1.53)	3.45 (2.98–3.99)	**<0.0001**
**Self-estimated nutrition knowledge**				
Low level	17.63 (14.05–22.13)	1.27 (1.06–1.52)	3.24 (2.71–3.88)	**<0.0001**
High level	13.22 (10.61–16.48)	1.30 (1.09–1.55)	3.48 (2.91–4.17)	**<0.0001**
**Grocery shopping frequency**				
Always	16.51 (12.68–21.49)	1.36 (1.11–1.67)	3.46 (2.80–4.26)	**<0.0001**
Sometimes	14.04 (10.79–18.27)	1.16 (0.94–1.43)	3.37 (2.73–4.17)	**<0.001**
Never	15.49 (11.48–20.90)	1.35 (1.05–1.73)	3.20 (2.51–4.09)	**<0.0001**
**Use of nutritional information during shopping**				
Always	11.47 (8.10–16.23)	1.10 (0.83–1.46)	2.96 (2.22–3.96)	**<0.0001**
Sometimes	14.77 (11.99–18.20)	1.33 (1.13–1.57)	3.37 (2.85–3.99)	**<0.0001**
Never	16.52 (10.99–24.82)	1.03 (0.74–1.42)	3.16 (2.29–4.36)	**<0.0001**
**Self-estimated diet quality**				
Low quality	18.16 (14.49–22.76)	1.33 (1.11–1.60)	3.45 (2.88–4.12)	**<0.0001**
High quality	12.80 (10.26–15.97)	1.23 (1.03–1.48)	3.27 (2.73–3.92)	**<0.0001**

The category of reference was “no label”. Data are presented as the OR (95% CI). Boldface indicates statistical significance (*p* < 0.05).

**Table 4:** Multivariate-adjusted HL and students’ characteristics according to the various clusters of preference for FOP labels.

Variable	Nutri-Score (*n* = 904)	RI (*n* = 446)	MTL (*n* = 604)	None (*n* = 306)	*p*-Value ^a^
**Health literacy**					**0.022**
Inadequate HL	18.5	12.6	14.7	15.0	
Problematic HL	46.4	47.3	51.5	43.8	
Sufficient HL	35.2	39.5	33.8	41.2	
Sex					**0.020**
Men	22.4	25.1	25.3	31.4	
Women	77.7	74.9	74.7	68.6	
**Age (years)**					**<0.0001**
≤21	51.3	52.9	42.7	38.2	
22–23	23.5	24.2	26.2	24.8	
≥24	25.2	22.9	31.1	36.9	
**Nationality**					**0.038**
Belgian	84.6	87.7	82.5	80.7	
Others	15.4	12.3	17.6	19.3	
**Higher education institution**					**<0.0001**
Non-university	39.7	34.5	28.3	34.0	
University	60.3	65.5	71.7	66.0	
**Education field**					0.086
Health	44.4	40.8	46.0	38.2	
Others	55.6	59.2	54.0	61.8	
**Smoking status**					0.833
Current smoker	17.8	18.2	15.4	15.4	
Former smoker	4.42	4.48	6.29	7.19	
Never smoker	77.8	77.4	78.3	77.5	
**Physical activity**					0.177
Competitive sport	12.0	13.2	11.3	11.4	
Leisure-time activity ≥4 h/week	22.7	31.2	29.6	31.4	
Leisure-time activity <4 h/week	43.9	40.8	43.0	36.6	
Sedentary behavior	19.3	13.0	14.7	18.3	
**BMI (kg/m^2^)**					0.108
≤18.5	67.7	71.3	72.2	70.9	
18.5–24.9	9.18	9.42	8.11	7.19	
25.0–29.9	15.3	14.4	17.1	18.6	
≥30.0	7.41	4.93	2.48	2.61	
**Household composition**					0.206
Alone	13.9	11.0	14.4	19.0	
With family	52.9	53.8	45.4	43.1	
Shared house	33.2	35.2	40.2	37.9	
**Perceived financial resources**					0.247
Difficult	26.6	25.1	22.9	21.9	
Easy	73.5	74.9	77.2	78.1	
**Self-estimated nutrition knowledge**					**<0.001**
Low level	60.7	45.1	42.2	41.5	
High level	39.3	54.9	57.8	58.5	
**Grocery shopping frequency**					**0.0007**
Always	34.4	33.9	43.1	42.2	
Sometimes	35.1	38.6	32.3	38.6	
Never	30.5	27.6	24.7	21.2	
**Use of nutritional information during shopping**					**<0.001**
Always	10.4	25.8	19.7	35.6	
Sometimes	56.0	55.8	61.4	46.7	
Never	33.6	18.4	18.9	17.7	
**Self-estimated diet quality**					**<0.001**
Low quality	58.7	48.4	47.2	42.2	
High quality	41.3	51.6	52.8	57.8	

Data are presented as the number (%). ^a^ *p*-Values from the multinomial logistic regression model. Boldface indicates statistical significance (*p* < 0.05).

## Data Availability

Not applicable.

## Supplementary Materials

The following supporting information can be downloaded at https://www.mdpi.com/article/10.3390/ijerph19148751/s1: Table S1. Questionnaire used to assess the perception of various FOP nutrition labels (French version and English translation); Table S2. Crude percentage of responses to the questions related to the perception of FOP labels; Table S3. Percentage (frequency) of clustering labels and reference labels according to perception questionnaire.

## References

[1] Afshin A., Sur P.J., Fay K.A., Cornaby L., Ferrara G., Salama J.S., Mullany E.C., Abate K.H., Abbafati C., GBD 2017 Diet Collaborators (2019). Health effects of dietary risks in 195 countries, 1990–2017: A systematic analysis for the Global Burden of Disease Study 2017. Lancet.

[2] World Health Organization (WHO), Food and Agriculture Organization of the United Nations (FAO) (2018). The Nutrition Challenge: Food System Solutions.

[3] Branca F., Lartey A., Oenema S., Aguayo V., Stordalen G.A., Richardson R., Arvelo M., Afshin A. (2019). Transforming the food system to fight non-communicable diseases. BMJ.

[4] United Nations (2017). UN Decade of Action on Nutrition (2016–2025) Work Programme.

[5] World Health Organization (2015). Regional Office for Europe. European Food and Nutrition Action Plan 2015–2020.

[6] Campos S., Doxey J., Hammond D. (2011). Nutrition labels on pre-packaged foods: A systematic review. Public Health Nutr..

[7] Grunert K.G., Wills J.M. (2007). A review of European research on consumer response to nutrition information on food labels. J. Public Health.

[8] Bridget K., Jewell J., World Health Organization. Regional Office for Europe (2018). What is the Evidence on the Policy Specifications, Development Processes and Effectiveness of Existing Front-of-Pack Food Labelling Policies in the WHO European Region Health Evidence Network (HEN) Synthesis Report 61.

[9] European Commission (2020). Report from the Commission to the European Parliament and the Council Regarding the Use of Additional Forms of Expression and Presentation of the Nutrition Declaration.

[10] Egnell M., Ducrot P., Touvier M., Allès B., Hercberg S., Kesse-Guyot E., Julia C. (2018). Objective understanding of Nutri-Score Front-Of-Package nutrition label according to individual characteristics of subjects: Comparisons with other format labels. PLoS ONE.

[11] Egnell M., Boutron I., Péneau S., Ducrot P., Touvier M., Galan P., Buscail C., Porcher R., Ravaud P., Hercberg S. (2021). Randomised controlled trial in an experimental online supermarket testing the effects of front-of-pack nutrition labelling on food purchasing intentions in a low-income popu-lation. BMJ Open.

[12] Mansfield E., Wahba R., De Grandpré E. (2020). Integrating a Health Literacy Lens into Nutrition Labelling Policy in Canada. Int. J. Environ. Res. Public Health.

[13] Gore F.M., Bloem P.J., Patton G.C., Ferguson J., Joseph V., Coffey C., Sawyer S.M., Mathers C.D. (2011). Global burden of disease in young people aged 10–24 years: A systematic analysis. Lancet.

[14] Kerver J.M., Yang E.J., Obayashi S., Bianchi L., Song W.O. (2006). Meal and snack patterns are associated with dietary intake of energy and nutrients in US adults. J. Am. Diet. Assoc..

[15] Pendergast F.J., Livingstone K.M., Worsley A., McNaughton S.A. (2016). Correlates of meal skipping in young adults: A systematic review. Int. J. Behav. Nutr. Phys. Act..

[16] Orfanos P., Naska A., Trichopoulos D., Slimani N., Ferrari P., van Bakel M., Deharveng G., Overvad K., Tjønneland A., Halkjær J. (2007). Eating out of home and its correlates in 10 European countries. The European Prospective Investigation into Cancer and Nutrition (EPIC) study. Public Health Nutr..

[17] Nelson M.C., Story M., Larson N.I., Neumark-Sztainer D., Lytle L.A. (2008). Emerging adulthood and college-aged youth: An overlooked age for weight-related behavior change. Obesity.

[18] Hernández-Jaña S., Huber-Pérez T., Palma-Leal X., Guerrero-Ibacache P., Campos-Nuñez V., Zavala-Crichton J.P., Jorquera-Aguilera C., Sadarangani K.P., Rodríguez-Rodríguez F., Cristi-Montero C. (2020). Effect of a Single Nutritional Intervention Previous to a Critical Period of Fat Gain in University Students with Overweight and Obesity: A Randomized Controlled Trial. Int. J. Environ. Res. Public Health.

[19] World Health Organization (WHO) (1995). Physical Status: The Use of and Interpretation of Anthropometry.

[20] Food Standards Agency (2007). Front of Pack Nutritional Signpost Labelling Technical Guidance.

[21] FoodDrinkEurope (2013). Guidance on the Provision of Food Information to Consumers—Regulation (EU) No. 1169/2011.

[22] Santé Publique France (2018). Conditions of Use of the “Nutri-Score” Logo.

[23] Ducrot P., Méjean C., Chantal J., Kesse Guyot E., Touvier M., Fezeu L., Hercberg S., Péneau S. (2015). Effectiveness of front-of-pack nutrition labels in french adults: Results from the nutrinet-santé cohort study. PLoS ONE.

[24] Ducrot P., Méjean C., Julia C., Kesse-Guyot E., Touvier M., Fezeu L.K., Hercberg S., Péneau S. (2015). Objective Understanding of Front-of-Package Nutrition Labels among Nutritionally At-Risk Individuals. Nutrients.

[25] Egnell M., Talati Z., Galan P., Andreeva V.A., Vandevijvere S., Gombaud M., Dréano-Trécant L., Hercberg S., Pettigrew S., Julia C. (2020). Objective understanding of the Nutri-score front-of-pack label by European consumers and its effect on food choices: An online experimental study. Int. J. Behav. Nutr. Phys. Act..

[26] De Ridder K., Bel S., Brocatus L., Cuypers K., Lebacq T., Moyersoen I., Ost C., Teppers E., Bel S., Tafforeau J. (2016). La Consommation Alimentaire.

[27] Vandevijvere S., De Vriese S., Huybrechts I., Moreau M., Temme E., De Henauw S., De Backer G., Kornitzer M., Leveque A., Van Oyen H. (2009). The gap between food-based dietary guidelines and usual food consumption in Belgium, 2004. Public Health Nutr..

[28] Julia C., Péneau S., Buscail C., Gonzalez R., Touvier M., Hercberg S., Kesse-Guyot E. (2017). Perception of different formats of front-of-pack nutrition labels according to sociodemographic, lifestyle and dietary factors in a French population: Cross-sectional study among the NutriNet-Santé cohort participants. BMJ Open.

[29] Pelikan J.M., Ganahl K., Broucke S.V.D., Sørensen K. (2019). Measuring health literacy in Europe: Introducing the European health literacy survey questionnaire (HLS-EU-Q). International Handbook of Health Literacy.

[30] Rouquette A., Nadot T., Labitrie P., Broucke S.V.D., Mancini J., Rigal L., Ringa V. (2018). Validity and measurement invariance across sex, age, and education level of the French short versions of the European Health Literacy Survey Questionnaire. PLoS ONE.

[31] Sørensen K., Pelikan J.M., Röthlin F., Ganahl K., Slonska Z., Doyle G., Fullam J., Kondilis B., Agrafiotis D., Uiters E. (2015). Health literacy in Europe: Comparative results of the European health literacy survey (HLS-EU). Eur. J. Public Health.

[32] Vandevijvere S., Vermote M., Egnell M., Galan P., Talati Z., Pettigrew S., Hercberg S., Julia C. (2020). Consumers’ food choices, understanding and perceptions in response to different front-of-pack nutrition labelling systems in Belgium: Results from an online experi-mental study. Arch. Public Health.

[33] Egnell M., Talati Z., Gombaud M., Galan P., Hercberg S., Pettigrew S., Julia C. (2019). Consumers’ Responses to Front-of-Pack Nutrition Labelling: Results from a Sample from The Netherlands. Nutrients.

[34] Paakkari L., Okan O. (2020). COVID-19: Health literacy is an underestimated problem. Lancet Public Health.

[35] Berkman N.D., Sheridan S.L., Donahue K.E., Halpern D.J., Crotty K. (2011). Low health literacy and health outcomes: An updated systematic review. Ann. Intern. Med..

[36] Bostock S., Steptoe A. (2012). Association between low functional health literacy and mortality in older adults: Longitudinal cohort study. BMJ.

[37] Cha E., Kim K.H., Lerner H.M., Dawkins C.R., Bello M.K., Umpierrez G., Dunbar S.B. (2014). Health literacy, self-efficacy, food label use, and diet in young adults. Am. J. Health Behav..

[38] Malloy-Weir L., Cooper M. (2017). Health literacy, literacy, numeracy and nutrition label understanding and use: A scoping review of the literature. J. Hum. Nutr. Diet..

[39] Taylor M.K., Sullivan D.K., Ellerbeck E.F., Gajewski B.J., Gibbs H.D. (2019). Nutrition literacy predicts adherence to healthy/unhealthy diet patterns in adults with a nutrition-related chronic condition. Public Health Nutr..

[40] VandenBosch J., Van den Broucke S., Vancorenland S., Avalosse H., Verniest R., Callens M. (2016). Health literacy and the use of healthcare services in Belgium. J. Epidemiol. Commun. Health.

[41] Avalosse H., Verniest R., Vancorenland S., De Cock S., Gérard F., Cornerotte S., Van den Broucke S. (2017). Littératie en santé (Health literacy) et sources d’information. Educ. Santé.

[42] Kühn L., Bachert P., Hildebrand C., Kunkel J., Reitermayer J., Wäsche H., Woll A. (2022). Health Literacy Among University Students: A Systematic Review of Cross-Sectional Studies. Front. Public Health.

[43] Juvinyà-Canal D., Suñer-Soler R., Porquet A.B., Vernay M., Blanchard H., Bertran-Noguer C. (2020). Health Literacy among Health and Social Care University Students. Int. J. Environ. Res. Public Health.

[44] Bröder J., Okan O., Bauer U., Schlupp S., Pinheiro P. (2019). Advancing perspectives on health literacy in childhood and youth. Health Promot. Int..

[45] Mansfield E.D., Ibanez D., Chen F., Chen E., De Grandpré E. (2020). Efficacy of “High in” Nutrient Specific Front of Package Labels—A Retail Experiment with Canadians of Varying Health Literacy Levels. Nutrients.

[46] Feunekes G.I.J., Gortemaker I.A., Willems A.A., Lion R., van den Kommer M. (2008). Front-of-pack nutrition labelling: Testing effec-tiveness of different nutrition labelling formats front-of-pack in four European countries. Appetite.

[47] Desbouys L., Méjean C., De Henauw S., Castetbon K. (2020). Socio-economic and cultural disparities in diet among adolescents and young adults: A systematic review. Public Health Nutr..

[48] Plotnikoff R.C., Costigan S.A., Williams R.L., Hutchesson M.J., Kennedy S.G., Robards S.L., Allen J., Collins C.E., Callister R., Germov J. (2015). Effectiveness of interventions targeting physical activity, nutrition and healthy weight for university and college students: A systematic review and meta-analysis. Int. J. Behav. Nutr. Phys. Act..

[49] Egnell M., Talati Z., Hercberg S., Pettigrew S., Julia C. (2018). Objective Understanding of Front-of-Package Nutrition Labels: An International Comparative Experimental Study across 12 Countries. Nutrients.

[50] Christoph M.J., An R. (2018). Effect of nutrition labels on dietary quality among college students: A systematic review and meta-analysis. Nutr. Rev..

[51] Cioffi C.E., Levitsky D.A., Pacanowski C.R., Bertz F. (2015). A nudge in a healthy direction. The effect of nutrition labels on food purchasing behaviors in university dining facilities. Appetite.

[52] Freedman M.R., Connors R. (2010). Point-of-purchase nutrition information influences food-purchasing behaviors of college students: A pilot study. J. Am. Diet. Assoc..

[53] Seward M.W., Block J.P., Chatterjee A. (2016). A Traffic-Light Label Intervention and Dietary Choices in College Cafeterias. Am. J. Public Health.

[54] Hamlin R.P., McNeill L.S., Moore V. (2015). The impact of front-of-pack nutrition labels on consumer product evaluation and choice: An experimental study. Public Health Nutr..

[55] Egnell M., Boutron I., Péneau S., Ducrot P., Touvier M., Galan P., Buscail C., Porcher R., Ravaud P., Hercberg S. (2019). Front-of-Pack Labeling and the Nutritional Quality of Students’ Food Purchases: A 3-Arm Randomized Controlled Trial. Am. J. Public Health.

[56] Al-Jawaldeh A., Rayner M., Julia C., Elmadfa I., Hammerich A., McColl K. (2020). Improving Nutrition Information in the Eastern Mediterranean Region: Implementation of Front-of-Pack Nutrition Labelling. Nutrients.

[57] Vadeboncoeur C., Foster C., Townsend N. (2018). Challenges of research recruitment in a university setting in England. Health Promot. Int..

[58] Drieskens S., Charafeddine R., Gisle L. (2018). Enquête de Santé 2018: Etat Nutritionnel.

[59] Gisle L., Demarest S., Drieskens S. (2018). Enquête de Santé 2018: Consommation de Tabac.

[60] Hauschildt K., Gwosć C., Schirmer H., Wartenbergh-Cras F. (2021). Social and Economic Conditions of Student Life in Europe—EUROSTUDENT VII Synopsis of Indicators 2018–2021.

