# Supplementary material for: Health Literacy and Its Associations with Understanding and Perception of Front-of-Package Nutrition Labels among Higher Education Students
Source: Int J Environ Res Public Health. 2022 Jul 19;19(14):8751. doi: 10.3390/ijerph19148751 (PMC9319699; doi:10.3390/ijerph19148751)
Supplement: Supplementary file 1 [file ijerph-19-08751-s001.zip › ijerph-1783792-supplementary.pdf]

Supplementary Materials

Supplementary Table S1. Questionnaire used to assess the perception of various FOP nutrition labels (French version and English translation).

Question (English translation)	Question (original French version)
This FOP label is the best to help me to choose healthier products	Ce logo aide à choisir des produits meilleurs pour la santé
I want to see this label on the front of packages	Je veux qu'il soit présent sur les emballages
This is my preferred label	C'est mon logo préféré
This is the label I appreciate the least	C'est le logo que j'aime le moins
This FOP label provides me with the information I need	Ce logo m'apporte l'information dont j'ai besoin
This FOP label is trustworthy	Ce logo m'inspire confiance
This FOP label provides reliable information	Ce logo permet d'avoir une information fiable
This FOP label is easy to identify	Ce logo est facile à repérer
This FOP label is easy to understand	Ce logo est facile à comprendre
This FOP label is quick to process	Ce logo permet d'avoir une information rapide
This FOP label is too complex to understand	Ce logo est trop compliqué à comprendre
This FOP label takes too long to understand	Ce logo est trop long à comprendre
This FOP label is guilt laden	Ce logo est culpabilisant

Supplementary Table S2. Crude percentage of responses to the questions related to the perception of FOP labels.

	**FOP labels**			
	Nutri-Score	RI	MTL	None
**This FOP label is the best to help me to choose healthier products (+)**	54.6	7.83	34.3	3.23
**I want to see this label on the front of packages (+)**	44.2	12.0	41.0	2.83
**This is my preferred label (+)**	54.0	9.07	31.7	5.27
**This is the label I appreciate the least (-)**	18.9	61.2	12.9	6.99
**This FOP label provides me with the information I need (+)**	18.9	19.5	58.5	3.19
**This FOP label is trustworthy (+)**	39.1	15.3	40.5	5.13
**This FOP label provides reliable information (+)**	8.81	25.3	61.1	4.87
**This FOP label is easy to identify (+)**	84.9	1.99	12.1	1.06
**This FOP label is easy to understand (+)**	81.5	2.70	14.3	1.55
**This FOP label is quick to process (+)**	83.9	1.95	13.0	1.11
**This FOP label is too complex to understand (-)**	3.94	56.5	13.1	26.5
**This FOP label takes too long to understand (-)**	1.99	65.4	13.6	19.0
**This FOP label is guilt laden (-)**	29.7	7.30	28.0	35.0

(+) Positively valenced item; (-) Negatively valenced item.

Supplementary Table S3. Percentage (frequency) of clustering labels and reference labels according to perception questionnaire.

	**Nutri-Score (n_1=904)**	**Reference Intakes (n_2=446)**	**Multiple Traffic Lights (n_3=604)**	**None (n_4=306)**
**This FOP label is the best to help me to choose healthier products**				
Nutri-Score	89.0%(810)	50.67%(226)	26.49%(160)	12.42%(38)
Reference Intakes	2.54%(23)	26.68%(119)	2.48%(15)	6.54%(20)
Multiple Traffic Lights	7.08%(64)	20.18%(90)	68.87%(416)	67.32%(206)
None	0.77%(7)	2.47%(11)	2.15%(13)	13.73%(42)
**I want to see this label on the front of packages**				
Nutri-Score	87.50%(791)	28.48%(127)	10.93%(66)	4.58%(14)
Reference Intakes	4.09%(37)	41.93%(187)	2.48%(15)	10.78%(33)
Multiple Traffic Lights	8.41%(76)	26.23%(117)	84.27%(509)	73.20%(224)
None	0%(0)	3.36%(15)	2.32%(14)	11.44%(35)
**This is my preferred label**				
Nutri-Score	96.90%(876)	39.69%(177)	25.99%(157)	3.27%(10)
Reference Intakes	0.55%(5)	34.75%(155)	2.15%(13)	10.46%(32)
Multiple Traffic Lights	2.32%(21)	18.83%(84)	67.72%(409)	66.01%(202)
None	0.22%(2)	6.73%(30)	4.14%(25)	20.26%(62)
**This is the label I appreciate the least**				
Nutri-Score	0.44%(4)	21.30%(95)	23.01%(139)	6.21%(19)
Reference Intakes	83.74%(757)	23.09%(103)	74.17%(448)	24.51%(75)
Multiple Traffic Lights	14.05%(127)	33.86%(151)	0.66%(4)	2.94%(9)
None	1.77%(16)	21.75%(97)	2.15%(13)	10.46%(32)
**This FOP label provides me with the information I need**				
Nutri-Score	37.39%(338)	10.09%(45)	5.96%(36)	2.29%(7)
Reference Intakes	10.73%(97)	54.71%(244)	5.46%(33)	21.90%(67)
Multiple Traffic Lights	50.44%(456)	32.96%(147)	86.92%(525)	63.07%(193)
None	1.44%(13)	2.24%(10)	1.66%(10)	12.75%(39)
**This FOP label is trustworthy**				
Nutri-Score	64.16%(580)	32.06%(143)	23.18%(140)	6.54%(20)
Reference Intakes	6.64%(60)	44.84%(200)	4.64%(28)	18.63%(57)
Multiple Traffic Lights	26.88%(243)	17.71%(79)	69.87%(422)	56.21%(172)
None	2.32%(21)	5.38%(24)	2.32%(14)	18.63%(57)
**This FOP label provides reliable information**				
Nutri-Score	18.03%(163)	5.83%(26)	1.16%(7)	0.98%(3)
Reference Intakes	17.15%(155)	59.64%(266)	10.43%(63)	28.43%(87)
Multiple Traffic Lights	62.72%(567)	29.37%(131)	84.93%(513)	55.23%(169)
None	2.10%(19)	5.16%(23)	3.48%(21)	15.36%(47)
**This FOP label is easy to identify**				
Nutri-Score	99.0%(895)	87.89%(392)	90.23%(545)	28.10%(86)
Reference Intakes	0.11%(1)	6.95%(31)	0.17%(1)	3.92%(12)
Multiple Traffic Lights	0.88%(8)	4.93%(22)	9.44%(57)	60.78%(186)
None	0%(0)	0.22%(1)	0.17%(1)	7.19%(22)
**This FOP label is easy to understand**				
Nutri-Score	97.12%(878)	83.86%(374)	87.09%(526)	20.92%(64)
Reference Intakes	0.22%(2)	8.74%(39)	0.33%(2)	5.88%(18)
Multiple Traffic Lights	2.65%(24)	6.50%(29)	12.25%(74)	63.73%(195)
None	0%(0)	0.90%(4)	0.33%(2)	9.48%(29)
**This FOP label is quick to process**				
Nutri-Score	95.91%(867)	85.43%(381)	89.40%(540)	35.62%(109)
Reference Intakes	0.33%(3)	7.17%(32)	0.17%(1)	2.61%(8)

	Nutri-Score (n_1=904)	Reference Intakes (n_2=446)	Multiple Traffic Lights (n_3=604)	None (n_4=306)
Multiple Traffic Lights	3.76%(34)	6.50%(29)	10.26%(62)	55.23%(169)
None	0%(0)	0.90%(4)	0.17%(1)	6.54%(20)
**This FOP label is too complex to understand**				
Nutri-Score	0.55%(5)	1.79%(8)	1.66%(10)	21.57%(66)
Reference Intakes	75.77%(685)	7.17%(32)	78.64%(475)	27.45%(84)
Multiple Traffic Lights	15.49%(140)	27.58%(123)	4.30%(26)	2.29%(7)
None	8.19%(74)	63.45%(283)	15.40%(93)	48.69%(149)
**This FOP label is too long to understand**				
Nutri-Score	0.44%(4)	1.57%(7)	0.33%(2)	10.46%(32)
Reference Intakes	81.64%(738)	20.18%(90)	87.75%(530)	38.89%(119)
Multiple Traffic Lights	15.38%(139)	28.03%(125)	5.13%(31)	4.25%(13)
None	2.54%(23)	50.22%(224)	6.79%(41)	46.41%(142)
**This FOP label is guilt laden**				
Nutri-Score	18.69%(169)	26.23%(117)	41.06%(248)	44.77%(137)
Reference Intakes	10.62%(96)	4.48%(20)	6.29%(38)	3.59%(11)
Multiple Traffic Lights	41.15%(372)	22.65%(101)	21.69%(131)	9.15%(28)
None	29.54%(267)	46.64%(208)	30.96%(187)	42.48%(130)
